# Air Quality Monitoring of the Post-Operative Recovery Room and Locations Surrounding Operating Theaters in a Medical Center in Taiwan

**DOI:** 10.1371/journal.pone.0061093

**Published:** 2013-04-03

**Authors:** Chin-Sheng Tang, Gwo-Hwa Wan

**Affiliations:** 1 Department of Public Health, College of Medicine, Fu Jen Catholic University, New Taipei City, Taiwan, R.O.C.; 2 Department of Respiratory Therapy, College of Medicine, Chang Gung University, Kwei-Shan, Tao-Yuan, Taiwan, R.O.C.; Chang Gung University, Taiwan

## Abstract

To prevent surgical site infection (SSI), the airborne microbial concentration in operating theaters must be reduced. The air quality in operating theaters and nearby areas is also important to healthcare workers. Therefore, this study assessed air quality in the post-operative recovery room, locations surrounding the operating theater area, and operating theaters in a medical center. Temperature, relative humidity (RH), and carbon dioxide (CO_2_), suspended particulate matter (PM), and bacterial concentrations were monitored weekly over one year. Measurement results reveal clear differences in air quality in different operating theater areas. The post-operative recovery room had significantly higher CO_2_ and bacterial concentrations than other locations. *Bacillus spp.*, *Micrococcus spp.*, and *Staphylococcus spp*. bacteria often existed in the operating theater area. Furthermore, *Acinetobacter spp*. was the main pathogen in the post-operative recovery room (18%) and traumatic surgery room (8%). The mixed effect models reveal a strong correlation between number of people in a space and high CO_2_ concentration after adjusting for sampling locations. In conclusion, air quality in the post-operative recovery room and operating theaters warrants attention, and merits long-term surveillance to protect both surgical patients and healthcare workers.

## Introduction

Hospital indoor air pollution is associated with inadequate building environments, including building materials, air conditioning systems, ventilation rates, and human factors, such as over-crowding in constrained spaces [1]–[3]. Evaluations of operating theater air quality assessed levels of particulate matter (PM), microbial agents, and volatile organic compounds (VOCs) [3]–[12]. Employees, patients, and visitors are significant sources of airborne microbes in hospitals.

The airborne microbial concentration is correlated with suspended PM sized 5–7μm [3], human activity, number of people in a space, and apparel worn by personnel in operating theaters [6]. The frequency with which people enter and exit operating theaters may also increase the quantity of microorganisms in indoor environments [10]. Airborne droplets frequently carry such bacteria as *Staphylococcus aureus* (*S. aureus*), *S epidemidis*, and gram-negative rods, which are common causes of postoperative wound infection [13]. Thus, the airborne microbial concentration must be reduced to prevent surgical site infection (SSI). Although safe airborne bacterial limits, such as 10 CFU/m^3^ [14] and 180 CFU/m^3^ [10], have been proposed, no international consensus exists regarding tolerable microbial levels in operating theaters.

To evaluate operating environments for surgical patients, a previous study evaluated variations in hospital indoor air quality (IAQ) indices in eight operating theaters at a medical center in northern Taiwan [3]. In addition to surgical patients, air quality in operating theater areas is also critical to healthcare workers. Reports have identified an increasing number of adverse health effects associated with time spent in mechanically ventilated buildings, typically in the workplace [15]–[17]. Symptoms are generally attributable either to exposure to a combination of substances or to increased individual susceptibility to low concentrations of contaminants [18].

Compared to operating theaters, more healthcare workers and surgical patients were present in post-operative recovery rooms. The IAQ of post-operative recovery rooms may be adversely affected by human activity. To date, most studies assessed air quality in hospital operating theaters, and did not measure IAQ in post-operative recovery rooms and areas surrounding operating theaters. Therefore, this study is the first to evaluate long-term variation in air quality in operating theater areas, including operating theaters, a post-operative recovery room, and other nearby locations in a medical center.

## Materials and Methods

### Sampling locations

**Figure 1:**
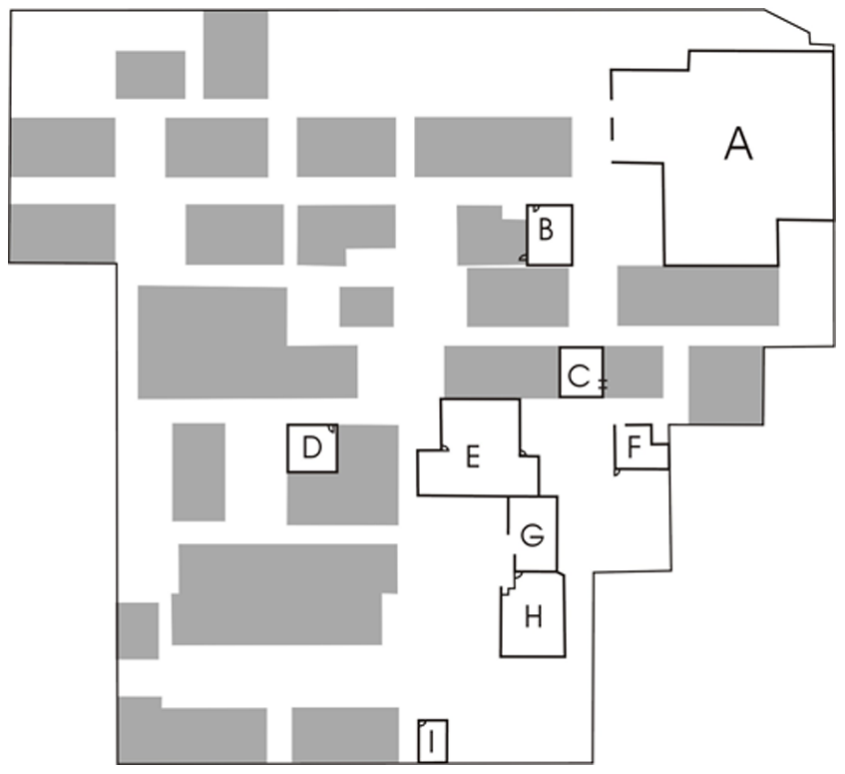
Diagram of operating theater area of a hospital. A: post-operative room; B: kidney transplant room; C: traumatic surgery room; D: liver transplant room; E: instrument room; F: office; G: supply washing room; H: restaurant; I: delivery room.

Permits for this study were obtained from Chang Gung Memorial Hospital. This study evaluated the IAQ in the operating theater area, including the operating theaters (kidney transplant room, liver transplant room, and traumatic surgery room), post-operative recovery room, and surrounding areas (instrument room, supply washing room, delivery room, restaurant, and office) in a medical center in northern Taiwan. Figure 1 shows the operating theater area. The post-operative recovery room (1,418.51 m^3^) is located in an open space and is adjacent to the kidney transplant room (121.80 m^3^). The instrument room (445.33 m^3^) is near the supply washing room (190.31 m^3^)_and restaurant (316.29 m^3^). The exit from the supply washing room is near the restaurant. The traumatic surgery room (113.10 m^3^) is near an office (105.31 m^3^). The liver transplant room (122.53 m^3^) and delivery room (60.9 m^3^) are located to the left and on the corner of the operating theater area, respectively. During the sampling period, indoor air was conditioned but not heated. A ceiling-mounted high efficiency particulate air (HEPA)-filtered laminar air flow with 15 air changes per hour (ACH) supplied the operating theater area, but did not serve the office and delivery room. The HEPA filters were changed annually. The office had only an exhaust air filter (ACH = 10/hour) which was cleaned at 4-month intervals, while the delivery room was fitted with an exhaust vent design without a filtration system (ACH = 12.5/hour).

### Air quality monitoring

Indoor air in the operating theater area was sampled once weekly (Monday to Friday) for one year. Air monitoring duration was 60 minutes in the morning (8 am to 12 pm) at each location. Air was sampled in operating theaters during operations. The air sampling instruments were placed approximately 1.5 m from the operating tables to avoid aseptic area contamination in the operating theaters during surgery, or were placed at the center of each sampling area, such as in the post-operative recovery room and locations surrounding the operating theater area._All instruments were positioned 1.2–1.5 meters above the floor to simulate the breathing zone of healthcare workers. The doors of operating theaters and locations surrounding the operating theater were always closed during the sampling period.

The evaluated air quality indices were air temperature, relative humidity (RH), and concentrations of CO_2_, suspended PM, and bacteria. Temperature, RH and CO_2_ concentration were determined using a digital psychrometer (TSI, Inc., Shoreview, MN, USA). The PM levels were measured using a portable dust monitor (Model 1.108; Grimm Labortechnik Ltd., Ainring, Germany). Bacterial concentrations were assessed using Andersen one-stage viable impactors (N6; Andersen Samplers, Atlanta, Georgia) with 20 mL of tryptic soy agar (TSA) at an airflow rate of 28.3 liters per minute (LPM) for 3 minutes. Duplicate bacteria samples were collected to ensure sampling accuracy, and bacterial samples were incubated at 30±1°C for 48±2 h, as recommended by Taiwan's Environmental Protection Agency (EPA) [19]. The positive hole conversion table [20] and sampled air volume were used to calculate the CFU/m^3^ values. The gram-positive bacteria (*Bacillus*, *Corynebacterium*, *Micrococcus*, *Staphylococcus*) and gram-negative bacteria (*Acinetobacter*, *Moraxella*, *Pseudomonas*, *Stenotrophomonas*) were identified biochemically.

### Statistical analysis

Data were analyzed by SPSS version 17.0 (SPSS, Inc., Chicago, USA). The significance level was *p*<0.05. The PM was classified as PM_10_ (aerodynamic diameter≤10 μm) and PM_2.5_ (aerodynamic diameter≤2.5 μm). The airborne bacterial concentration was log-normally distributed. The IAQ indices at different locations in the operating theater area were compared using the one-way analysis of variance test with Scheffe *post hoc* comparisons for normally distributed data. A chi-square test was used to determine differences in isolation rates of airborne bacteria at different sampling locations in the operating theater area. Pearson correlation analysis was applied to identify the relationship between two continuous variables with normally distributed data. Such mixed-effect models have the advantages of the adjustment of invariant variables by fixed-effect models and the consideration of individual differences by random-effect models [21]. In this study, mixed-effect models were used to identify factors, such as air temperature, RH, number of people in a space, and different sampling locations, influencing levels of CO_2_, PM_2.5_, and airborne bacteria, and quantify their respective correlation strength.

## Results

Analytical results demonstrate that all air quality indices differed markedly at different locations surrounding the operating theater (*p*<0.01). Mean air temperature in the operating theater area was 18.9–22.3°C (Table 1). Temperatures measured at locations surrounding the operating theater, such as the post-operative recovery room, instrument room, supply washing room, delivery room, and restaurant, were significantly higher than those in the operating theaters (*p*<0.05). Humidity was 63.6–70.7% at different locations in the operating theater area. The highest and lowest CO_2_ concentrations were in the post-operative recovery room (651 ppm) and delivery room (406.1 ppm), respectively. Both the post-operative recovery room and office (580 ppm) had significantly higher CO_2_ concentrations than the operating theaters (*p*<0.05). Moreover, the PM_10_ (40.1 μg/m^3^) and PM_2.5_ (6.5 μg/m^3^) concentrations in the delivery room were significantly higher than those at other locations in the operating theater area (*p*<0.01). Both the supply washing room and restaurant had higher PM_10_ and PM_2.5_ concentrations than the operating theaters. The highest bacterial concentrations were in the post-operative recovery room (383.5 CFU/m^3^), supply washing room (373.7 CFU/m^3^), and restaurant (270.8 CFU/m^3^), and clearly exceeding those in the operating theaters (92.0–87.19 CFU/m^3^, *p*<0.05). The highest density of people (19) was in the post-operative recovery room in the operating theater area.

**Table 1:** Air quality measurement of the operating theater area.

Air quality indices	Locations surrounding operating theaters						Operating theaters		
	POR	IR	SWR	DR	Restaurant	Office	KTR	TR	LTR
Temperature, °C	21.1±1.5^*†‡^	20.6±1.5^*‡^	22.3±1.7^*†‡^	21.9±2.6^*†‡^	20.9±1.4^*‡^	20.0±1.3	18.9±1.4	19.7±1.4	19.1±1.1
RH, %	66.2±3.7	63.8±5.5^*†‡^	63.6±5.0^*†‡^	69.9±6.8	70.7±4.7	65.8±3.7^*^	70.0±3.8	68.3±3.1	68.6±5.4
CO_2_, ppm	651.0±97.7^*†‡^	529.4±84.7	517.1±74.2	406.1±65.3^*†‡^	521.0±78.3	580.0±90.2^†‡^	545.5±80.4	480.4±71.7	498.2±80.0
PM_10_, μg/m^3^	19.7±8.2	13.3±8.7	40.1±18.6^*†‡^	40.2±26.1^*†‡^	35.7±26.3^*†‡^	31.8±19.4	12.9±8.4	20.7±11.4	15.2±8.2
PM_2.5_, μg/m^3^	3.5±2.0	1.2±1.0	6.5±4.8	16.5±15.4^*†‡^	9.1±9.2^*†‡^	1.4±0.8	1.1±0.6	1.7±1.4	1.0±0.5
Bacteria, CFU/m^3^	383.5 (2.1)^*†‡^	106.9 (2.0)^†‡^	373.7 (1.6)^*†‡^	141.5 (2.2)^*‡^	270.8 (1.8)^*†‡^	182.2 (1.8)^*‡^	92.0 (2.3)	144.5 (2.0)	87.19 (1.9)
number of people in a space, n	19±6^*†‡^	3±1^*^	4±2^*^	1±1^*†‡^	6±5	2±2^*‡^	6±2	4±2	5±2

Data were represented as mean±sd or geometric mean (GSD). *: *p*<0.05 compared with KTR; †: *p*<0.05 compared with TR; ‡: *p*<0.05 compared with LTR.

POR: post-operative recovery room; IR: instrument room; SWR: supply washing room; DR: delivery room; KTR: kidney transplant room; TR: traumatic surgery room; LTR: liver transplant room.

The isolation rate of bacterial species varied markedly among different locations within the operating theater area (Table 2). The predominant isolated genus included gram-positive bacteria such as *Bacillus spp.* (including *B. brevis*, *B. cereus*, and *B. megaterium*), *Corynebacterium spp*., *Micrococcus spp.* (including *M. luteus and M. lylae*), *and Staphylococcus spp.* (including *S. aureus*, *S. capitis*, *S. epidermidis*, *S. haemolyticus*, *S. hominis*, and *S. saprophyticus*), and gram-negative bacteria such as *Acinetobacter spp. (including A. baumannii* and *A. lwoffii*), *Moraxella spp.*, *Pseudomonas spp.*, and *Stenotrophomonas spp*. The isolation rates of gram-positive bacteria exceeded those of gram-negative bacteria in the operating theater area. Gram-positive bacteria *Bacillus spp.*, *Micrococcus spp*., and *Staphylococcus spp*. were identified frequently in the operating theater area. *Bacillus spp.* was the main bacteria detected in the post-operative recovery room (32%) and delivery room (47%). *Micrococcus spp.* was the main bacteria detected in the instrument room (34%), supply washing room (33%), and kidney transplantation room (38%). The level of *Staphylococcus spp.* (45%) was highest in the traumatic surgery room. *Stenotrophomonas spp*. existed only in the kidney transplant room (4%). Additionally, *Acinetobacter spp.* was the predominant species in the post-operative recovery room (18%), instrument room (10%), and traumatic surgery room (8%). Significant differences existed in isolation rates of *Staphylococcus spp.* (*p* = 0.01) and *Acinetobacter spp.* (*p*<0.01) at different sampling locations in the operating theater area.

**Table 2:** Isolation rates of airborne bacteria (%)† in the operating theater area.

Microorganisms	Locations surrounding operating theaters												Operating theaters						*p* value‡
	POR		IR		SWR		DR		Restaurant		Office		KTR		TR		LTR		
Gram-positive bacteria																			
*Bacillus spp*	26	(32%)	11	(27%)	9	(23%)	22	(47%)	7	(17%)	13	(29%)	18	(32%)	7	(18%)	10	(23%)	0.07
*Corynebacterium spp*	1	(1%)	1	(2%)	1	(2%)	4	(9%)	1	(2%)	3	(7%)	2	(4%)	2	(5%)	1	(2%)	0.58
*Micrococcus spp*	21	(26%)	14	(34%)	13	(33%)	10	(21%)	12	(30%)	13	(29%)	21	(38%)	8	(21%)	13	(29%)	0.65
*Staphylococcus spp*	16	(20%)	10	(25%)	13	(33%)	6	(12%)	15	(37%)	15	(33%)	10	(18%)	17	(45%)	16	(36%)	0.01
Gram-negative bacteria																			
*Acinetobacter spp*	15	(18%)	4	(10%)	1	(2%)	1	(2%)	2	(5%)	0	(0%)	0	(0%)	3	(8%)	0	(0%)	<0.01
*Moraxella spp*	1	(1%)	0	(0%)	1	(2%)	0	(0%)	0	(0%)	1	(2%)	1	(2%)	0	(0%)	1	(2%)	0.88
*Pseudomonas spp*	1	(1%)	0	(0%)	0	(0%)	0	(0%)	1	(2%)	0	(0%)	0	(0%)	0	(0%)	1	(2%)	0.69
*Stenotrophomonas spp*	0	(0%)	0	(0%)	0	(0%)	0	(0%)	0	(0%)	0	(0%)	2	(4%)	0	(0%)	0	(0%)	0.09
Others	1	(1%)	1	(2%)	2	(5%)	4	(9%)	3	(7%)	0	(0%)	1	(2%)	1	(3%)	2	(4%)	0.32

† : the number of specific isolated microorganism was divided by the total number of isolated microorganisms;

‡ : chi-square test.

POR: post-operative room; IR: instrument room; SWR: supply washing room; DR: delivery room; KTR: kidney transplant room; TR: traumatic surgery room; LTR: liver transplant room.

Number of people in a space and CO_2_ concentration (r = 0.61, *p*<0.01), and number of people in a space and bacterial concentration (r = 0.36, *p*<0.01) in the operating theater area were positively correlated (Table 3). The CO_2_ concentration was moderately associated with the bacterial concentration (r = 0.38, *p*<0.01). Additionally, the bacterial concentration was moderately associated with PM_10_ level (r = 0.42, *p*<0.01) and weakly related to PM_2.5_ level (r = 0.19, *p*<0.01), respectively. The mixed-effect model shows a strong relationship between number of people in a space and high CO_2_ concentration (β = 0.09, *p*<0.01) after adjusting for sampling location (Table 4). However, no significant relationship existed between number of people in a space and bacterial concentrations after adjusting for air temperature, RH, and sampling location. Most sampling locations had a lower CO_2_ concentration than that in the office in the operating theater area. However, the post-operative recovery room had a higher CO_2_ concentration. Adjusting for the influence of number of people in a space, significantly higher PM_2.5_ levels existed in the four sampling locations–the post-operative recovery room, supply washing room, delivery room, and restaurant–than in the office (*p*<0.01). Additionally, the bacterial concentration in the supply washing room significantly exceeded that in the office, after adjusting for air temperature, RH, and number of people in the space (*p* = 0.04).

**Table 3:** Correlation matrix of environmental factors in the operating theater area of a hospital.

Environmental factors	(1)	(2)	(3)	(4)	(5)	(6)	(7)
(1) Temperature, °C	1						
(2) Humidity, %	−0.183^**^	1					
(3) CO_2_, ppm	−0.070	−0.100*	1				
(4) PM_10_, μg/m^3^	0.163^**^	0.171^**^	0.004	1			
(5) PM_2.5_, μg/m^3^	0.179^**^	0.259^**^	−0.190^**^	0.726^**^	1		
(6) Bacteria, CFU/m^3†^	0.301^**^	−0.062	0.376^**^	0.415^**^	0.191^**^	1	
(7) Number of people, n	−0.009	0.014	0.607^**^	−0.043	−0.133*	0.356^**^	1

* : *p*<0.05; **: *p*<0.01; ^†^: The bacterial concentration was calculated by geometric transformation.

**Table 4:** Associations between CO_2_/PM_2.5_/bacterial concentrations and environmental variables in the operating theater area.

Environmental variables	CO_2_ concentration			PM_2.5_ level			Bacterial concentration †		
	β	SE	*p* value	β	SE	*p* value	β	SE	*p* value
Intercept	5.10	0.20	<0.01	0.82	0.29	<0.01	0.27	0.20	0.17
Temperature	-		-	-		-	−0.005	0.04	0.89
Relative humidity	-		-	-		-	0.02	0.03	0.57
Number of persons in a space	0.09	0.02	<0.01	−0.002	0.05	0.98	−0.003	0.01	0.76
Sampling location‡									
POR	0.80	0.28	<0.01	2.13	0.38	<0.01	0.05	0.08	0.50
IR	−0.54	0.28	0.06	−0.29	0.39	0.46	0.01	0.08	0.93
SWR	−0.75	0.28	0.01	2.29	0.39	<0.01	0.17	0.08	0.04
DR	−1.59	0.28	<0.01	2.46	0.39	<0.01	−0.04	0.08	0.65
Restaurant	−0.72	0.28	0.01	3.58	0.39	<0.01	−0.02	0.08	0.76
KTR	−0.71	0.29	0.02	−0.28	0.42	0.50	0.03	0.09	0.73
TR	−1.15	0.28	<0.01	0.35	0.40	0.38	−0.06	0.08	0.47
LTR	−0.99	0.28	<0.01	−0.26	0.41	0.53	0.06	0.08	0.45

† : The bacterial concentration was calculated by geometric transformation;

‡ : office was used as a control location. SE: standard error.

POR: post-operative room; IR: instrument room; SWR: supply washing room; DR: delivery room; KTR: kidney transplant room; TR: traumatic surgery room; LTR: liver transplant room.

## Discussion

This is the first study in Taiwan to apply air quality indices to an operating theater area, including the post-operative recovery room and surrounding areas in a medical center, to document differences in air quality. Until recently, no international consensus existed regarding the best method and frequency of air sampling, and the tolerable bioburden in operating theater areas. Thus, the interval between sampling was determined by each institution using available means [22].

Long-term air monitoring reveals that mean CO_2_ concentration in the post-operative recovery room was highest. This study also found that the CO_2_ concentration was positively correlated with number of people in the operating theater area. A high number of people, say, over 19 in the post-operative recovery room may be associated with high measured CO_2_ concentrations. Researchers have recommended a high air exchange rate (20/hr) to achieve airborne bacterial concentrations of 50–150 CFU/m^3^ [22]. However, few countries have set a bacterial limit in conventionally ventilated operating theaters. In the United Kingdom, the bacterial limit is 35 CFU/m^3^ for an empty operating theater, while that for an active theater is 180 CFU/m^3^ for an average 5-min period [23]. Findings obtained by this study show that the post-operative recovery room had the highest airborne bacterial concentration. Additionally, 9.8–35.3% of bacterial samples from operating theaters had concentrations exceeding the limit of 180 CFU/m^3^, as set by the UK National Health Service. Therefore, bioaerosol exposure for surgical patients and healthcare workers in the post-operative recovery room warrants further attention.

In this study, correlation analysis results indicate that the number of people in the operating theater area was correlated with bacterial concentrations in that area. However, no significant correlation existed between number of people in a space and the airborne bacterial concentration after adjusting for temperature, RH, and sampling location. We hypothesize that variations in the airborne bacterial concentration depend on sampling locations with different functions in the operating theater area of a hospital. Thus, hospitals should consider controlling the number of occupant (estimated maximum occupancy of 20 persons/1000 ft^2^) and increasing outdoor air requirements (15 cfm/person) in operating theater areas to achieve an acceptable IAQ [24]. Additionally, appropriate staff dress and discipline can minimize the spread of bacteria from healthcare personnel and reduce airborne microbial contamination [22]. Furthermore, the airborne bacterial concentration in the operating theater area was positively associated with PM_10_ and PM_2.5_ levels in that area. The use of cleanroom technology standards based on the presence of air particles can be considered routine procedure for monitoring the distribution of bacterial concentrations in operating theater areas [22].

*Acinetobacter baumannii*, *Pseudomonas aeruginosa*, *Klebsiella pneumoniae*, and *Staphylococcus aureus* are commonly associated with nosocomial respiratory tract infections in Taiwanese hospitals [25]. This investigation detected pathogenic bacteria, such as *Staphylococcus spp*., *Acinetobacter spp*., and *Pseudomonas spp*., which were implicated in nosocomial infections in the operating theater area. Detection rates (12%–45%) for *Staphylococcus aureus* were highest in all locations in the operating theater area. *Bacillus spp.*, *Micrococcus spp*., and *Staphylococcus spp*. bacteria were common in the operating theater area. This result is in agreement with that of a previous study [26]. The distribution of microbial species in the operating theater area, particularly in the post-operative recovery room and operating theaters, warrants attention from hospital environmental safety and health departments to reduce exposure risk to surgical patients and healthcare workers.

A limitation of this study is that IAQ measurements other than bacterial concentration were taken for only 1 h; future studies can extend the duration of air sampling to improve the IAQ evaluation. Different factors affected environmental sampling results, which cannot be expected to remain constant over time [22]. Long-term monitoring of air quality in operating theater areas is necessary, particularly in the post-operative recovery room and operating theaters in hospitals, to provide a safe environment for surgical patients and working environment for hospital employees. Further evaluation is required to identify potential IAQ problems that result from healthcare procedures and equipment. A previous study indicated that ventilation systems were a source of infection; in some cases, systems spread infectious pathogens [27]. Thus, cleaning and maintenance frequency for ventilation systems in operating theater areas can be adjusted based on system operating hours and number of occupants in an area.

In conclusion, air quality of operating theater areas, particularly that in post-operative recovery rooms and operating theaters deserves attention, and requires long-term surveillance by environmental safety and health departments in hospitals to protect both surgical patients and healthcare workers.
